# Long Noncoding RNA Expression Profile in BV2 Microglial Cells Exposed to Lipopolysaccharide

**DOI:** 10.1155/2019/5387407

**Published:** 2019-06-11

**Authors:** Yajuan Li, Qingmin Li, Cunjuan Wang, Shengde Li, Lingzhi Yu

**Affiliations:** ^1^Department of Pain Management, Jinan Central Hospital, Shandong University, Jinan 250013, China; ^2^Department of Anesthesiology, Taian City Central Hospital, Taian 271000, China; ^3^Department of Neurosurgery, Taian City Central Hospital, Taian 271000, China; ^4^Department of Children Rehabilitation, W. F. Maternal and Child Health Hospital, Weifang 261011, China

## Abstract

Neuropathic pain, which is one of the most common forms of chronic pain, seriously increases healthcare costs and impairs patients' quality of life with an incidence of 7–10% worldwide. Microglia cell activation plays a key role in the progression of neuropathic pain. Better understanding of novel molecules modulating microglia cell activation and these underlying functions will extremely benefit the exploration of new treatment. Recent studies suggested long noncoding RNAs may be involved in neuropathic pain. However, its underlying functions and mechanisms in microglia cell activation remain unclear. To identify the differentially expressed lncRNAs and predict their functions in the progression of microglia cell activation, GSE103156 was analyzed using integrated bioinformatics methods. The expression levels of selected lncRNAs and mRNAs were determined by real-time PCR. In the present study, a total of 56 lncRNAs and 298 mRNAs were significantly differentially expressed. The differentially expressed mRNAs were mainly enriched in NF-kappa B signaling pathway, TNF signaling pathway, Toll-like receptor signaling pathway, and NOD-like receptor signaling pathway. The top 10 hub genes were Tnf, Il6, Stat1, Cxcl10, Il1b, Tlr2, Irf1, Ccl2, Irf7, and Ccl5 in the PPI network. Our results showed that Gm8989, Gm8979, and AV051173 may be involved in the progression of microglia cell activation. Taken together, our findings suggest that lots of lncRNAs may be involved in BV2 microglia cell activation in vitro. The findings may provide relevant information for the development of promising targets for the microglial cells activation of neuropathic pain in vivo in the future.

## 1. Introduction

Neuropathic pain, which is one of the most common forms of chronic pain [1, 2], seriously increased healthcare costs and impairs patients' quality of life with an incidence of 7–10% worldwide [3, 4]. Moreover, there are no effective treatments for patients with neuropathic pain. To design new prophylactic and therapeutic strategies, more studies are needed to explore the pathogenesis of neuropathic pain. Neuropathic pain often results from traumatic, infectious, chemical, metabolic, or cancerous impairments [3–5], in which significant features are hyperalgesia, allodynia, and spontaneous burning pain. Although the pathogenesis of neuropathic pain is obscure, microglia cell activation has been shown to be essential for neuropathic pain [6, 7]. A recent study has shown that spinal microglia activation was induced by peripheral nerve injury and contributed to central sensitization [8].

Considerable advances have been made in high-throughput technology for identifying microglial factors in neuropathic pain [9–11] in the past decade. However, the mechanisms of microglia activation-induced neuropathic pain are still poorly understood.

In general, ncRNAs do not encode functional proteins. It has shown that lncRNAs participate in most essential biological processes at the levels of posttranscription, transcription, and epigenetics [12–15]. Recent research has shown that some lncRNAs may be involved in the pathophysiology of neuropathic pain. Xiuli Zhao et al. reported that Kcna2 antisense RNA was an endogenous trigger in the progression of neuropathic pain [16]. Differentially expressed lncRNAs were identified in SNI-induced neuropathic pain using high-throughput sequencing techniques, identifying a series of potential therapeutic targets of neuropathic pain [17]. However, the potential functions and mechanisms of lncRNAs in microglia cell activation remain incompletely understood.

In this study, we investigated the expression profile of lncRNAs and mRNAs in BV2 microglial cells stimulated with LPS and predicted the potential functions and mechanisms of differentially expressed lncRNAs. Due to opportunities to identify novel promising targets of BV2 microglial cells activation, our results may provide relevant information for future study of microglial cells activation of neuropathic pain in vivo.

## 2. Materials and Methods

### 2.1. Cell Culture, LPS Treatment, and RNA Isolation

BV2 microglial cells were purchased from ATCC (Manassas, VA, USA) and cultured according to the manufacturer's instructions. BV2 microglial cells were stimulated for 24 h with LPS (1 *μ*g/ml) or vehicle control (HBSS) in DMEM high glucose media containing 2.5% FBS. Total RNA was isolated and identified as previously described.

### 2.2. Microarray Data

Raw data of GSE103156 (Affymetrix Mouse Gene 2.1 ST Array) were downloaded from the GEO data repository [18, 19]. In the present study, three BV2 control samples and three BV2 LPS samples were used for bioinformatic analysis.

### 2.3. Identification of Differentially Expressed lncRNAs and mRNAs

Using Transcriptome Analysis Console (TAC) 4.01 (Affymetrix, Santa Clara, CA, USA), differentially expressed lncRNAs and differentially expressed genes (DEGs) were identified. 2.0-fold or greater and an adjusted* p* value < 0.01 were selected as a threshold.

### 2.4. Gene Ontology (GO) and Pathway Enrichment Analyses

The Database for Annotation, Visualization and Integrated Discovery (DAVID; http://david.ncifcrf.gov) (version 6.8) was used to analyze the biological function of differentially expressed genes [20–24].* p* value < 0.05 was selected as a threshold.

### 2.5. Protein-Protein Interactions (PPI) Network and Module Analysis

The interactions of DEGs were predicted by STRING online database (http://string-db.org, version 10.5) [25]. PPI network was drawn by Cytoscape (version 3.7.1) [26] and its plugin (MCODE [27] and CytoNCA [28]).

### 2.6. Transcription Factor (TF) Regulatory Network Analysis

IRegulon plugin in Cytoscape was used to predict TFs of selected DEGs [29]. Normalized enrichment score (NES) > 10 was used as the thresholds.

### 2.7. LncRNA-mRNA Coexpression Network

LncRNA-mRNA coexpression network was constructed to analyze the interactions between lncRNA and mRNA by weighted correlation network analysis (WGCNA) as described previously [30].

### 2.8. Real-Time PCR

SuperReal PreMix Plus (Tiangen, Beijing, China) was used to perform real-time PCR in the AriaMx Real-time PCR System (Agilent Technologies, Palo Alto, CA). The reaction conditions were as follows: incubation at 95°C for 10 min, followed by 40 cycles of 95°C for 15 s, 61°C for 20 s, and 72°C for 30 s. The 2-ΔΔCt method was used to calculate the relative expression levels of selected lncRNAs and mRNA normalizing to GAPDH levels.

### 2.9. Statistical Analysis

All data were expressed as the mean ± SEM. Unpaired Student's t-test for parametric data and Mann-Whitney's U-test for nonparametric data were utilized for comparisons between 2 groups. GraphPad Prism 7.04 (GraphPad Software, San Diego, CA, USA) was used for all statistical analyses.* p* value < 0.05 was considered statistically significant.

## 3. Results

### 3.1. Identification of Differentially Expressed lncRNAs and mRNAs

**Figure 1:**
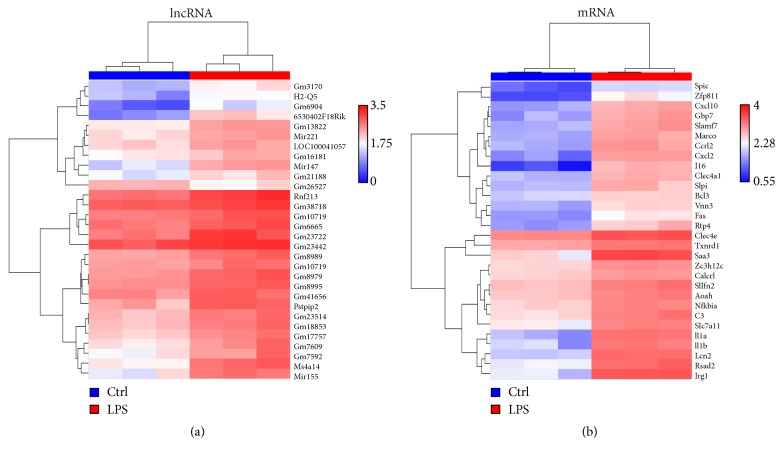
Hierarchical clustering of differentially expressed lncRNAs (a) and mRNAs (b). Red and blue columns refer to high and low relative expression, respectively.

In total, 56 lncRNAs and 298 mRNAs were significantly differentially expressed in BV2 microglial cells exposed to LPS. The top 30 most significantly differentially expressed lncRNAs (Figure 1(a)) and mRNAs (Figure 1(b)) were shown on heat map.

### 3.2. GO and Pathway Enrichment Analyses

**Figure 2:**
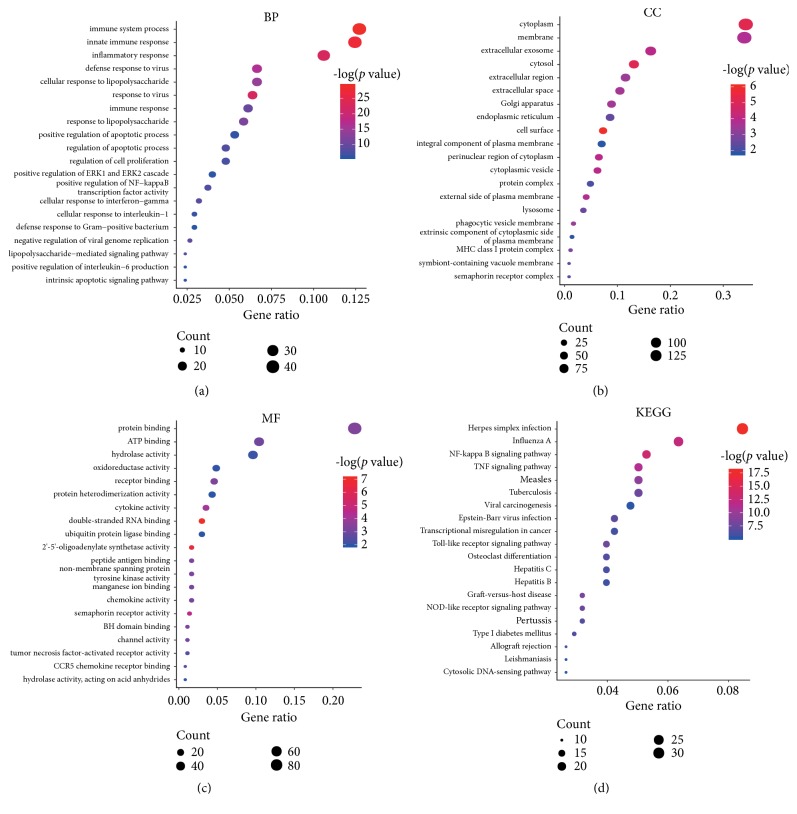
GO and KEGG enrichment analyses of differentially expressed mRNAs (Top 20,* p *< 0.05). GO functional analysis includes three categories: biological process (BP) (a), cellular component (CC), (b) and molecular function (MF) (c). Red to green colors indicate high to low -log (*p* value) levels. Point size indicates the number of differentially expressed genes in the corresponding pathway.

DEGs were mainly enriched in the following functions: immune system process, innate immune response, inflammatory response, and response to lipopolysaccharide (Figures 2(a)–2(c)). Our results also suggested that DEGs were mainly enriched in the following pathways: Herpes simplex infection, NF-kappa B signaling pathway, TNF signaling pathway, Toll-like receptor signaling pathway, and NOD-like receptor signaling pathway (Figure 2(d)).

### 3.3. PPI Network Analysis

**Figure 3:**
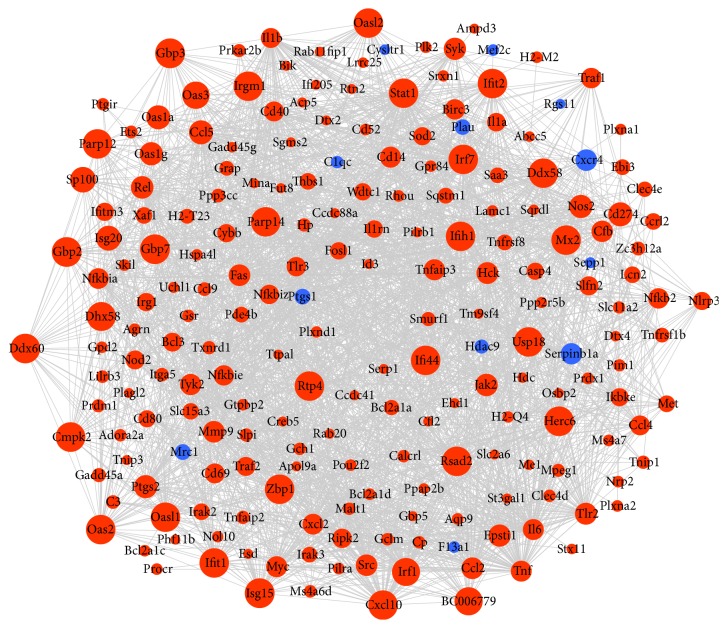
PPI network of 210 differentially expressed mRNAs with 1842 interaction pairs. Blue dots and red dots indicate downregulated and upregulated mRNAs, respectively. Circle size indicates the node degree.

To identify hub genes of microglia cell activation, we used STRING to look for interactions of DEGs in BV2 microglial cells stimulated with LPS. In the present study, we constructed a PPI network of 210 nodes and 1842 interaction pairs (Figure 3). In the PPI network, the top 10 most significantly hub genes were Tnf, Il6, Stat1, Cxcl10, Il1b, Tlr2, Irf1, Ccl2, Irf7, and Ccl5.

### 3.4. TF Regulatory Network Analysis

**Figure 4:**
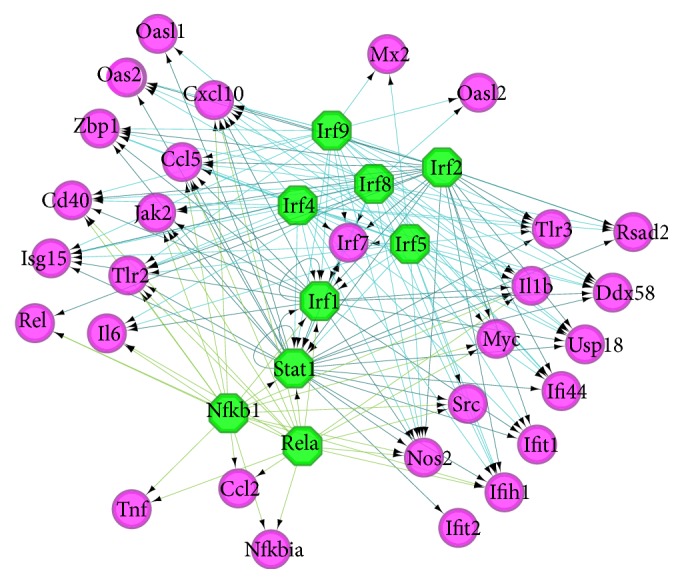
TF regulatory network of the top 30 hub genes in the PPI network. Green octagons represent TFs, and purple circles represent their correlated mRNAs.

The TFs of the top 30 hub genes in the PPI network were predicted. With NES > 10, nine TFs (Irf1, Irf2, Irf4, Irf5, Irf8, Irf9, Stat1, Nfkb1, and Rela) were predicted in the TF regulatory network (Figure 4).

### 3.5. LncRNA-mRNA Coexpression Network

**Figure 5:**
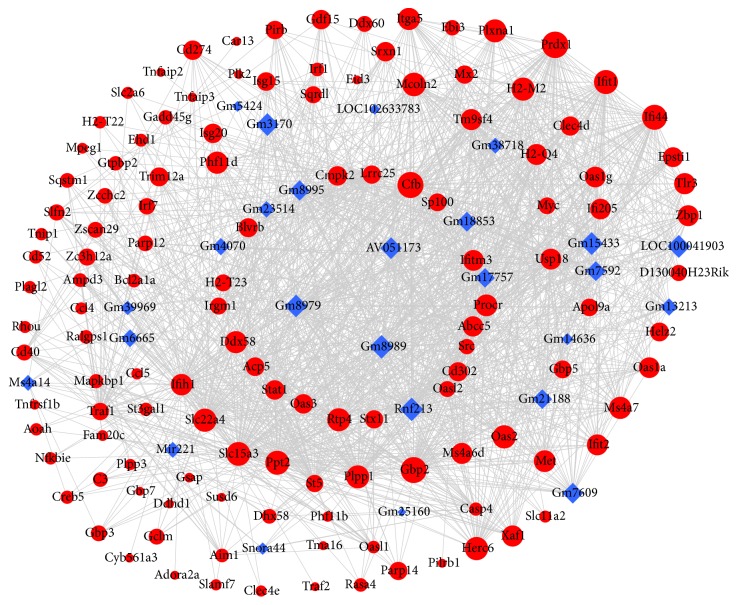
Coexpression network of 26 differentially expressed lncRNAs and 127 interacting differentially expressed mRNAs. The diamonds with blue represent lncRNAs; the circles with red represent their correlated mRNAs. Circle size indicates the node degree.

We constructed the lncRNA-mRNA coexpression network of 26 differentially expressed lncRNAs and 127 interacting DEGs to predict the functions and mechanisms of differentially expressed lncRNAs in BV2 microglial cells treated with LPS (Figure 5). The top 5 hub lncRNAs were Gm8989, Gm8979, Gm8995, AV051173, and Gm7609 in the coexpression network.

### 3.6. Real-Time PCR

**Figure 6:**
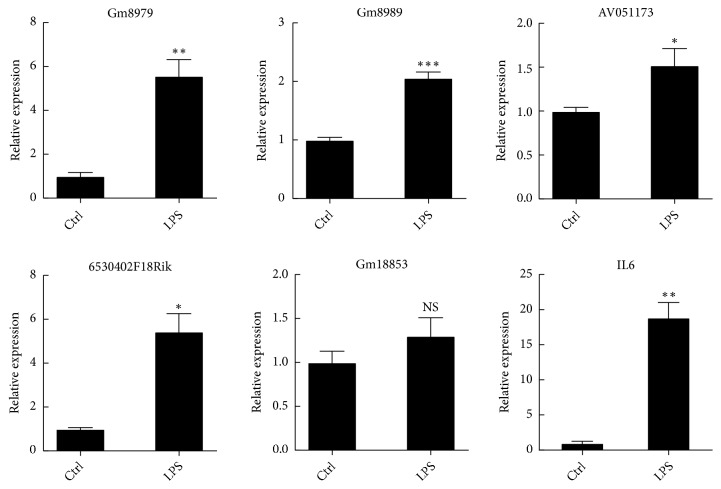
Real-time PCR of five lncRNAs and IL6 mRNA in BV2 microglial cells exposed to LPS. All data are means ± SEM (n=3), Student's* t*-test. *∗ P *< 0.05; *∗∗ P *< 0.01; *∗∗∗ P *< 0.001; NS, not significant.

Five lncRNAs (6530402F18Rik, AV051173, Gm8979, Gm8989, and Gm18853) and IL6 mRNA were selected to determine their relative expression levels by real-time PCR (Figure 6). Our results showed that Gm8979, Gm8989, AV051173, 6530402F18Rik, and IL6 were upregulated. The expression of Gm18853 showed no significant change.

## 4. Discussion

Neuropathic pain seriously increases healthcare costs and impairs patients' quality of life with an incidence of 7–10% worldwide [3, 4]. Microglia cell activation is essential to the progression of neuropathic pain in vivo [6, 7]. The research of microglia cell activation provides opportunities to reveal the molecular and cellular basis of neuropathic pain [6–8].

It has shown that lncRNAs participate in most essential biological processes at the levels of posttranscription, transcription, and epigenetics [12–15, 31]. Over the past decade, there have been several lncRNA transcriptome researches in chronic pain. Differentially expressed lncRNAs were identified in SNI-induced neuropathic pain using high-throughput sequencing techniques, revealing a series of potential therapeutic targets of neuropathic pain [17]. Unlike the previous study, which made the spinal cord as a whole, we downloaded and analysed raw data of GSE103156, which established microglia cell activation model by stimulating BV2 microglial cells with LPS. In addition to common properties of immortalized cell lines (e.g., increased proliferation and adherence), BV2 cells retain most crucial functions of microglia in immune response and inflammation [32, 33]. BV2 cells stimulated with LPS in vitro resemble the microglial response in vivo to some extent.

In the present study, we identified 56 differentially expressed lncRNAs and 298 DEGs in BV2 microglial cells stimulated with LPS. DEGs were mainly enriched in the following functions: immune system process, innate immune response, inflammatory response, and response to lipopolysaccharide. Pathway analysis results showed that DEGs mainly involved in Herpes simplex infection, NF-kappa B signaling pathway, TNF signaling pathway, Toll-like receptor signaling pathway, and NOD-like receptor signaling pathway. The experimental model was successfully developed since the mRNA expression levels of Il6, Il1a, Il1b, Tnf, and Cxcl10 increased in BV2 microglial cells stimulated with LPS [34, 35]. The expression levels of IL6 mRNAs in the microarray were similar to those detected by real-time PCR.

PPI network analysis results showed that many differentially expressed genes, such as Irf1, Irf7, Stat1, and Tlr2, acted as hub genes in microglia cell activation. Recent studies have revealed a crosstalk between Tlr2 and microglia cell activation [6, 36]. Although further experimental validation was needed, our results suggested new directions for future experimental research. We predicted 9 TFs (Irf1, Irf2, Irf4, Irf5, Irf8, Irf9, Stat1, Nfkb1, and Rela) mapping to 32 hug genes in PPI network. Notably, IRF1, IRF9, Stat1, and Nfkb1 were significantly upregulated in BV2 microglial cells exposed to LPS in the microarray analysis results.

Many studies have indicated that interferon regulatory factor (IRF) family was involved in the pathophysiology of microglial activation and neuropathic pain [37–39]. IRF8, interacted with IRF1 and IRF5, played an important regulatory role in the progression of neuropathic pain [40–42]. Recent studies have shown that Stat1 and Nfkb1 were closely related to neuropathic pain [43–46]. However, the regulatory mechanisms of the IRF family and other transcriptional factors in microglia cell activation should be further studied to determine their therapeutic effects in neuropathic pain in vivo.

Based on fold change and degree in the lncRNA-mRNA coexpression network, five lncRNAs (AV051173, Gm8979, Gm8989, 6530402F18Rik, and Gm18853) were selected to evaluate the expression levels using real-time PCR. The relative expression levels of selected lncRNAs, except for Gm18853, were consistent with these trends in the microarray. The variation between real-time PCR and microarray may relate to differences between the methods [47].

LncRNAs often transcribed together with their adjacent or overlapping target genes and regulate their expression through cis-regulation. Integrated bioinformatics analysis showed that AV051173 was coexpressed with Prdx1, with its location next to the Prdx1 gene on the same chromosome. Notably, Prdx1 was upregulated in BV2 microglial cells exposed to LPS. Prdx 1 regulated NF-*κ*B-mediated microglial activation as an antioxidant [48]. AV051173 might be involved in microglia cell activation by cis-regulatory role in the expression of Prdx1.

In contrast to cis-regulating lncRNAs, trans-regulating lncRNAs regulate gene expression far away from the site of primary locus of transcription [49, 50]. Gm8979 and Gm8989 are Gvin1 pseudogene, located on Chromosome 7. In the lncRNA-mRNA coexpression network, Gm8979 and Gm8989 were significantly coexpressed with mRNAs (Stat1 and Tlr3) via trans-acting mechanism. Stat1 and TLR3 played an important regulatory role in BV2 cell activation [44, 51]. Gm8979 and Gm8989 might be involved in microglial cell activation by regulating the expression of Stat1 and Tlr3 through a trans-acting mechanism.

Despite the results obtained above, there were some limitations in this study. Firstly, even with the similarity to primary microglia, BV2 microglial cells contain oncogenes which render them different from primary microglia in some ways, such as proliferation, adhesion, and the variance of morphologies. Secondary, the vitro model of microglia cell activation has limitations because the condition is encompassed by multiple cell types and responses in vivo. Therefore, it needs to be considered with caution about the association between the findings and the microglial cells activation of neuropathic pain in vivo.

## 5. Conclusions

In conclusion, we downloaded raw data of GSE103156 from the GEO data repository and identified differentially expressed lncRNAs and DEGs in BV2 microglia cell activation. The findings suggested that differentially expressed lncRNAs may regulate the expression of target genes acting as cis-acting or trans-acting factors. The findings may provide relevant information for the development of promising targets for the microglial cells activation of neuropathic pain in vivo in the future.

## Data Availability

The data used to support the findings of this study are available from the corresponding author upon request.
